# Critical Analysis of Data Leakage in WiFi CSI-Based Human Action Recognition Using CNNs

**DOI:** 10.3390/s24103159

**Published:** 2024-05-16

**Authors:** Domonkos Varga

**Affiliations:** Nokia Bell Labs, 1082 Budapest, Hungary; domonkos.varga@nokia-bell-labs.com

**Keywords:** WiFi CSI, human action recognition, convolutional neural networks, data leakage, machine learning

## Abstract

WiFi Channel State Information (CSI)-based human action recognition using convolutional neural networks (CNNs) has emerged as a promising approach for non-intrusive activity monitoring. However, the integrity and reliability of the reported performance metrics are susceptible to data leakage, wherein information from the test set inadvertently influences the training process, leading to inflated accuracy rates. In this paper, we conduct a critical analysis of a notable IEEE Sensors Journal study on WiFi CSI-based human action recognition, uncovering instances of data leakage resulting from the absence of subject-based data partitioning. Empirical investigation corroborates the lack of exclusivity of individuals across dataset partitions, underscoring the importance of rigorous data management practices. Furthermore, we demonstrate that employing data partitioning with respect to humans results in significantly lower precision rates than the reported 99.9% precision, highlighting the exaggerated nature of the original findings. Such inflated results could potentially discourage other researchers and impede progress in the field by fostering a sense of complacency.

## 1. Introduction

In recent years, the utilization of WiFi channel state information (CSI) for human action recognition has garnered significant attention due to its non-intrusive nature and potential for application in various fields such as healthcare monitoring [1], security surveillance [2], and human–computer interaction [3]. Leveraging advanced machine learning techniques, particularly convolutional neural networks (CNNs), researchers have reported remarkable accuracy rates in identifying human actions solely based on WiFi CSI data.

Among the plethora of studies in this domain, a notable IEEE Sensors Journal paper [4] claimed an exceptional accuracy rate of 99% of WiFi CSI-based human action recognition using CNNs. Such a high accuracy rate holds immense promise for practical applications, potentially revolutionizing how human actions are monitored and analyzed in various contexts. However, beneath the surface of these seemingly groundbreaking results lies a critical concern: data leakage. Data leakage, often overlooked or underestimated, poses a significant threat to the integrity and reliability of machine learning models. In the context of WiFi CSI-based human action recognition, data leakage can occur when information from the test set inadvertently leaks into the training process, leading to inflated performance metrics and misleading conclusions.

This study makes several significant contributions to the field of WiFi CSI-based human action recognition using machine/deep learning, particularly in identifying and addressing data leakage.

- Detection of data leakage: Our study successfully detects and analyzes instances of data leakage within the experimental methodology of a prominent IEEE Sensors Journal publication [4]. By meticulously examining the data partitioning methods, performance metrics, and model behavior reported in the original study, we identify inconsistencies and anomalies indicative of data leakage.
- Empirical validation: Through empirical validation and meticulous scrutiny of the dataset and experimental procedures, we provide concrete evidence to support our assertion of data leakage in the original study.
- Recommendations for mitigation: Building upon our findings, we propose practical recommendations for mitigating data leakage and enhancing the integrity of WiFi CSI-based human action recognition using machine/deep learning.

This paper is structured as follows: In Section 2, we provide a brief overview of the background literature related to WiFi CSI-based human action recognition and the significance of addressing data leakage. Section 3 outlines the methodology employed in the original study, followed by a detailed examination of the experimental setup and results in Section 4. In Section 5, we present our critical analysis of the findings, highlighting instances of data leakage and their impact on the reported accuracy rates. Finally, we conclude the paper in Section 6 by summarizing our key findings, discussing the implications of data leakage in WiFi CSI-based human action recognition, and suggesting avenues for future research.

## 2. Related Work

### 2.1. Human Action Recognition Based on WiFi Channel State Information

Human action recognition (HAR) algorithms may utilize different data modalities [5], such as RGB image [6], skeleton [7], depth [8], infrared [9], point cloud [10], event stream [11], audio [12], acceleration [13], radar [14], and WiFi [15]. Further, a significant amount of studies focuses on the fusion of different modalities for HAR [16,17,18,19]. In this section, we review methods utilizing WiFi CSI for HAR. WiFi CSI refers to the data obtained by monitoring the changes in the wireless channel characteristics between a transmitter (such as a WiFi access point) and a receiver (such as a WiFi-enabled device). This information includes various parameters such as signal strength, signal phase, signal-to-noise ratio (SNR), and other channel properties. WiFi CSI is collected by specialized hardware such as software-defined radios (SDRs) or WiFi chipsets that support CSI reporting. It provides detailed insights into the wireless channel’s behavior, allowing for advanced signal processing techniques and analysis. Researchers and engineers use WiFi CSI for various purposes, such as channel estimation [20], localization [21], gesture recognition [22], activity recognition [23], or wireless networking research [24]. WiFi CSI can be used for human action recognition due to the following reasons:

- Effect of human actions on wireless signals: Human actions, such as gestures or movements, can cause changes in the wireless channel characteristics due to blockage, reflection, or absorption of the WiFi signals. These changes are reflected in the WiFi CSI measurements.
- Distinctive patterns in CSI: Different human actions result in characteristic patterns in the WiFi CSI data. For example, a specific gesture may cause a sudden drop or fluctuation in signal strength or phase, which can be detected and recognized through signal processing techniques.
- Machine learning algorithms: Advanced machine learning algorithms can be trained to recognize specific human actions based on patterns observed in WiFi CSI data. By collecting the labeled data of WiFi CSI corresponding to different human actions, classifiers can be trained to accurately recognize and classify these actions in real time.

Due to the availability of WiFi signals, many WiFi CSI based systems for HAR have been proposed in the literature recently. Recent studies on WiFi CSI-based human action recognition have introduced various methods to improve accuracy and address different challenges. For instance, Wang et al. [25] discuss a device-free fall detection system called WiFall, which leverages wireless signal propagation models and CSI to detect falls without the need for wearable devices. Specifically, it employs the time variability and special diversity of CSI. The system consists of a two-phase detection architecture: a local outlier factor-based algorithm to identify abnormal CSI series and activity classification using one-class support vector machine (SVM) [26] to distinguish falls from other human activities. In contrast, the detection of large-scale human movements was the goal in the WiSee [27], WiTrack [28], Wi-Vi [29], and E-eyes [15] projects. Specifically, Pu et al. [27] extracted human gesture and motion information from wireless signals using the Doppler shift property, which results in a pattern of frequency shifts at the wireless receiver when a user performs a gesture or moves. WiSee also maps these Doppler shifts to gestures by leveraging the continuous nature of human gestures and classifying them using a binary pattern-matching algorithm. Additionally, the system works effectively in the presence of multiple users by utilizing multiple-input multiple-output capabilities to focus on gestures and motion from a specific user. In the WiTrack [28] project, 3D human motion tracking was carried out based on radio frequency reflections from a human body. Further, WiTrack can also provide a coarse tracking of larger human parts, such as legs or arms. In the Wi-Vi [29] project, Adib et al. demonstrated that the detection of human movements is also possible behind walls and doors in a closed room. In the E-eyes [15] project, researchers introduced a low-cost system for identifying activities in the home environments using WiFi access points and devices. The system uses the cumulative moving variance of CSI samples to determine the presence of walking or in-place activities. For activity identification, it employs dynamic time warping [30] for walking activities and the earth mover’s distance [31] for in-place activities, comparing the testing CSI measurements to known activity profiles. In [32], Halperin et al. issued a publicly available tool for WiFi collection and processing according to 802.11n standard [33] for a specific Intel chipset. Alternatives for Atheros chip sets were provided by Xie et al. [34] and Tsakalaki and Schäfer [35].

Recent studies in the field of WiFi CSI-based human action recognition has heavily utilized different deep-learning architectures and techniques. For instance, Chen et al. [36] applied Bi-LSTM and attention mechanism to learn from CSI amplitude and phase characteristics. Similarly, Guo et al. [37] applied an LSTM network but it was combined with a CNN. In contrast, Zhang et al. [38] proposed adversative auto-encoder networks for CSI signal security. Jiang et al.’s [39] consisted of three main components: the feature extractor, activity recognizer, and domain discriminator. The feature extractor, a CNN, collaborates with the activity recognizer to recognize human activities and simultaneously aims to fool the domain discriminator to learn environment/subject-independent representations. The domain discriminator is designed to identify the environment where activities are recorded, forcing the feature extractor to produce environment-independent activity features. Zhu et al. [40] combined casual and dilated convolution to implement a temporal convolution network.

### 2.2. Data Leakage in Machine Learning Models

Research on data leakage in machine learning models spans a variety of contexts and methodologies [41]. Data leakage occurs when information from the test set unintentionally influences the training process, leading to inflated performance metrics and misleading conclusions. This can happen due to various reasons, including improper data partitioning, feature engineering, or preprocessing techniques. Data leakage undermines the generalizability of machine learning models, as they may learn spurious correlations rather than genuine patterns in the data. The consequences of data leakage may extend beyond the realm of machine learning algorithms. In fields such as healthcare, finance, and security, relying on models affected by data leakage can have dire consequences [42,43,44]. Misguided decisions based on inaccurate predictions can result in financial losses, compromised patient care, or breaches in security protocols [45,46,47]. In [48], Poldrack et al. pointed out that a number of papers in the field of neuroimaging may have suffered from data leakage by performing dimensionality reduction across the whole dataset before the train/test split. Kapoor and Narayanan [49] identified eight types of leakage, i.e., not having a separate test set, preprocessing on the training and test sets, feature selection jointly on the training and test sets, duplicate data points, illegitimate features, temporal leakage, non-independence between the training and test sets, and sampling bias. Further, the authors identified 329 studies across 17 fields containing data leakage.

## 3. Methodology

**Figure 1:**
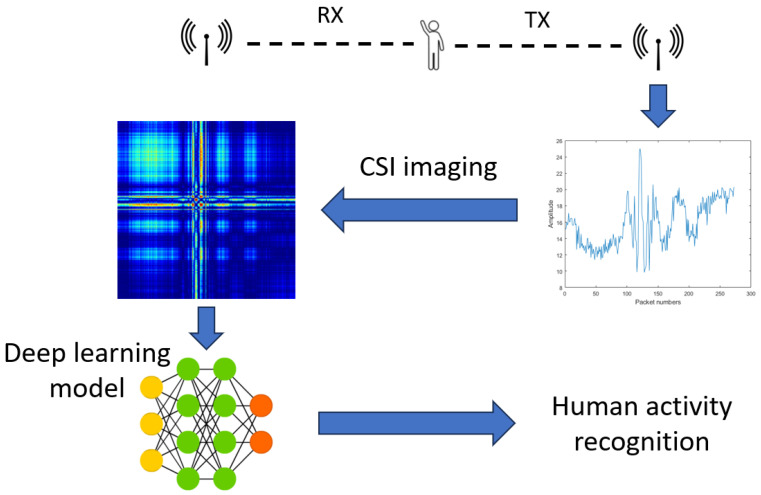
Framework for WiFi CSI based HAR.

The HAR framework, ImgFi [4], addresses the challenges of recognizing human activities using WiFi CSI data by converting the information into images and applying a CNN as an image classificator for improved performance—as illustrated in Figure 1. By introducing five CSI imaging approaches, i.e., recurrence plot (RP) transformation [50], Gramian angular summation field (GASF) transformation [51], Gramian angular difference field (GADF) transformation [51], Markov transition field (MTF) transformation [52], and short-time Fourier transformation (STFT) [53], the framework demonstrates the advantage and limitation of each method in processing CSI imaging. Since the authors of [4] found that RP slightly outperforms GASF, GADF, and STFT and significantly outperform MTF, the authors applied RP as CSI imaging in *ImgFi*. It follows that RP was also applied to our analysis and reimplementation of *ImgFi*. RP transformation is a method used in time series analysis and nonlinear dynamics to visualize the recurrence behavior of a dynamical system [54]. It is particularly useful for detecting hidden patterns, periodicities, and other nonlinear structures within time series data.

### 3.1. Structure of ImgFi

**Figure 2:**
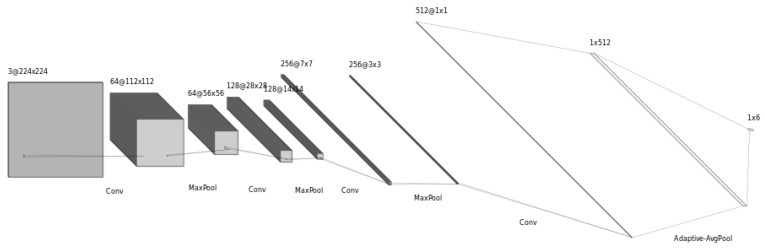
Structure of ImgFi model [4] which consists of four convolutional layers and a fully-connected layer which is the final classifier in this structure. Further, batch normalization and max pooling were applied between every two convolutional layers.

The structure of the proposed ImgFi CNN model is depicted in Figure 2. This model was reimplemented in PyTorch [55] strictly following the authors’ description. As it can be seen in Figure 2, ImgFi consists of four convolutional layers and a fully connected layer which is the final classifier in this structure. Furthermore, the authors implemented batch normalization [56], activation—rectified linear units (ReLU)—, and max pooling layer between every two convolutional layers.

Besides the implementation of *ImgFi*, the authors [4] gave the performance of several on ImageNet database pretrained CNNs, such as Shufflenet [57], VGG19 [58], ResNet18 [59], and ResNet50 [59]. ShuffleNet [57] is a CNN architecture designed for efficient computation and memory usage, particularly suited for mobile and embedded devices. It employs the concept of group convolutions and channel shuffling to significantly reduce the computational complexity while maintaining high accuracy in image classification tasks. VGG19 [58] is composed of 19 layers, including convolutional layers followed by max-pooling layers, and topped with fully connected layers. The network architecture consists of alternating convolutional layers with small 3×3 filters and max-pooling layers with 2×2 filters. This structure allows VGG19 to capture complex patterns at different scales in the input images. The last few layers of VGG19 are fully connected layers responsible for high-level reasoning and classification. ResNet18 and ResNet50 [59] are both CNN architectures developed by Microsoft Research as part of the ResNet (residual network) family. They are designed to address the vanishing gradient problem encountered in very deep neural networks by introducing skip connections or shortcuts. The main characteristics of the examined CNNs are summarized in Table 1.

Since a detailed description of the finetuning process of these architectures is not given in the original publication [4], we briefly summarize here our applied procedure. First, fully-connected layers were removed from the pretrained CNN models because these are specific to the original task (ImageNet classification). Second, we added new fully connected layers on top of the convolutional base. These layers will be specific to our new task. Specifically, the number of nodes in the final fully connected layer should match the number of classes in our dataset.

### 3.2. Detected Data Leakage

**Figure 3:**
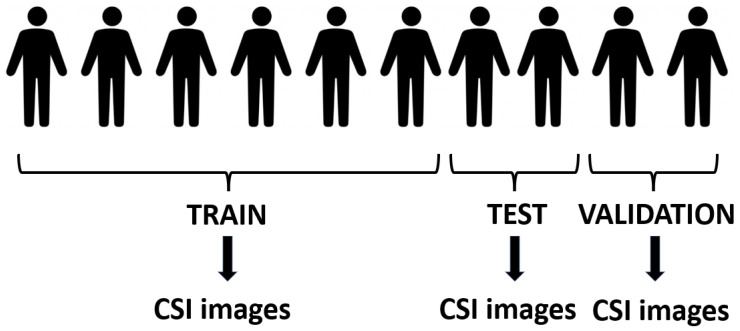
Illustration of dataset split with respect to humans. Humans are exclusively allocated to either training, validation, or test sets, ensuring independence and preventing data leakage between partitions.

**Figure 4:**
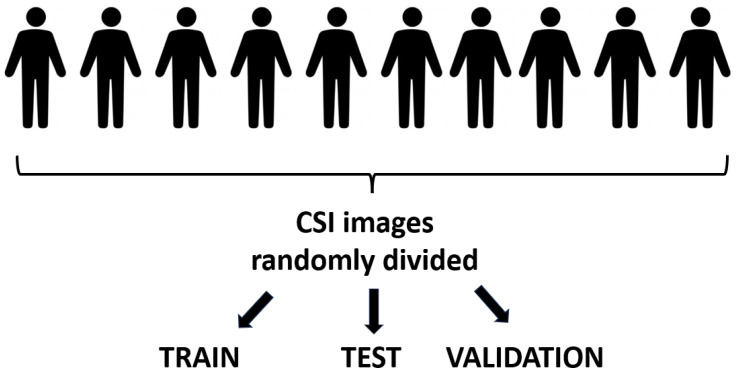
Illustration of dataset split without respect to humans. Humans are not allocated exclusively to training, validation, or test sets, but CSI images are generated first and then these CSI images are randomly divided into training, validation, and test sets. As a consequence, samples from one human can very easily occur both in the training and test sets leading to data leakage.

The authors [4] have not dedicated a separate section or paragraph on dataset partition in their paper. However, they claimed an exceptional accuracy rate of 99%. In the experimental results, we empirically corroborate that the authors divided the dataset in their methodology without respect to individual subjects, a crucial oversight that undermines the integrity of the study. By neglecting to divide the dataset with respect to the individuals, the authors inadvertently introduced a significant source of data leakage. The absence of subject-based data partitioning in the training, validation, and test sets raises concerns regarding the potential overlap of CSI images originating from the same individual across these sets. This oversight introduces a significant risk of data leakage, as CSI samples from a given individual may inadvertently influence the training process and subsequently inflate the reported accuracy metrics. Without a systematic approach to ensure the exclusivity of subjects across the dataset partitions, the study’s findings are susceptible to biases and inaccuracies, undermining the credibility of the proposed methodology. For clarity, Figure 3 and Figure 4 illustrate the differences between dataset partitioning with respect to and without respect to humans, respectively. As depicted in Figure 3, partitioning with respect to humans ensures the exclusivity of subjects across training, validation, and test sets, avoiding the risk of data leakage. In contrast, Figure 4 demonstrates the dataset division without respect to humans. In this incorrect strategy, CSI images are generated first (the exact number of CSI channels available in a Wi-Fi system may vary depending on the specific implementation and hardware capabilities [61]. Subsequently, these CSI images are randomly divided into training, validation, and test sets. As a consequence, samples from one human can very easily occur both in the training and test sets leading to data leakage. Why does dataset partition without respect to humans cause data leakage? Let us consider a scenario where we develop a product for WiFi-based human action recognition and intend to sell it to a market in another country. In this new market, the demographic composition, cultural norms, and individual identities will undoubtedly differ from those in our local setting. If our model is trained without proper consideration for subject-based partitioning, it may inadvertently learn patterns specific to the individuals in our local dataset. Consequently, when deployed in a different country where the population characteristics vary, the model’s performance may degrade significantly. By partitioning the data with respect to humans, we ensure that the model learns generalizable patterns of human actions rather than relying on idiosyncratic features of specific individuals. This approach not only enhances the model’s adaptability to diverse populations but also promotes its robustness and reliability across different cultural contexts.

Empirical analysis confirms that the authors generated CSI images first, and subsequently partitioned the dataset without respect to human identities. Through meticulous examination of the dataset, it became evident that CSI images originating from the same individual were distributed across the training, validation, and test sets without proper isolation. This critical oversight in the data partitioning process introduces a significant risk of data leakage, as features specific to individual subjects may inadvertently influence model training and evaluation, leading to inflated performance metrics. By empirically corroborating the lack of subject-based data partitioning, we underscore the necessity of adhering to rigorous data management protocols to ensure the integrity and reliability of machine learning studies.

## 4. Results

### 4.1. Data Details and Training

In the ImgFi study [4], the authors used three publicly available datasets, i.e., WiAR [62], SAR [63], Widar3.0 [64], and an own database to test the proposed CNN-based solutions. Since WiAR [62] and Widar3.0 [64] are the largest among the used publicly available datasets, we opted to use WiAR and Widar3.0 for the demonstration of the detected data leakage issue. Table 2 gives information on the action labels and the dataset size.

In Table 3, the parameter setting, which was used in the training of *ImgFi* and in the finetuning of the pre-trained CNN models, can be seen. Unlike [4], the WiFi CSI-based HAR dataset was divided with respect to the human subjects into training, validation, and test sets. As a consequence, CSI data originating from the same individual cannot be distributed across the train, validation, and test sets. As already mentioned, we empirically corroborate that the data split was carried out without respect to the human subjects in [4] which results in exceptionally high classification accuracy.

### 4.2. Evaluation Metrics

In our analysis to ensure correctness, we have used exactly the same evaluation metrics as proposed in [4]. Since the applied dataset was balanced, accuracy, precision, recall, and F1 were determined for each human action label and subsequently their arithmetic mean was given. In the examined classification problem, accuracy for each category can be expressed using the terms true positive (TP), true negative (TN), false positive (FP), and false negative (FN) as

(1) $$Acc=\frac{TP+TN}{TP+TN+FP+FN}.$$

Precision and recall for each category can be given as

(2) $$Prec=\frac{TP}{TP+FP},$$

(3) $$Recall=\frac{TP}{TP+FN}.$$

Similarly, F1 for each category can be expressed as

(4) $$F1=\frac{2\cdot Prec\cdot Recall}{Prec+Recall}.$$

If the number of categories is denoted by *N*, accuracy, precision, and F1 for all categories can be determined as follows:

(5) $$Macro\_Acc=\frac{1}{N}\sum_{i=1}^{N}Acc,$$

(6) $$Macro\_Prec=\frac{1}{N}\sum_{i=1}^{N}Prec,$$

(7) $$Macro\_Recall=\frac{1}{N}\sum_{i=1}^{N}Recall,$$

(8) $$Macro\_F1=\frac{1}{N}\sum_{i=1}^{N}F1.$$

### 4.3. Numerical Results

The numerical results are illustrated in Table 4 and Table 5. Specifically, the results reported in [4] and the results of the two dataset partition protocols—without respect to and with respect to humans—are compared. Our findings revealed that while the results of *ImgFi*’s retraining without respect to humans are slightly lower than the reported 99.9% precision, there are potential factors that may contribute to this discrepancy. One possible explanation could be the application of a data augmentation technique in [4] which was not reported in the paper. Additionally, it is important to note that our retraining process aimed to replicate the methodology of the original study to the best of our ability, but minor variations in the implementation may have influenced the final performance metrics. Despite the slight disparity in results, our analysis underscores the significance of implementing rigorous data management practices, such as subject-based partitioning, to ensure the integrity and reliability of machine learning studies in the domain of WiFi CSI-based human action recognition. Upon retraining the model with data partitioning carried out with respect to humans, we observed a notable decrease in the performance metrics compared to the reported results in the IEEE Sensors Journal paper [4]. Specifically, our retraining yielded a precision of 23.4%, recall of 22.8%, and F1 score of 22.0% on WiAR and precision of 47.4%, recall of 45.6%, and F1 score of 43.9% on Widar3.0, respectively. These findings highlight the critical role of proper data partitioning in ensuring the integrity and reliability of model evaluation. While the decrease in performance may be discouraging, it underscores the necessity of adhering to rigorous data management practices to obtain more accurate and generalizable results.

**Figure 5:**
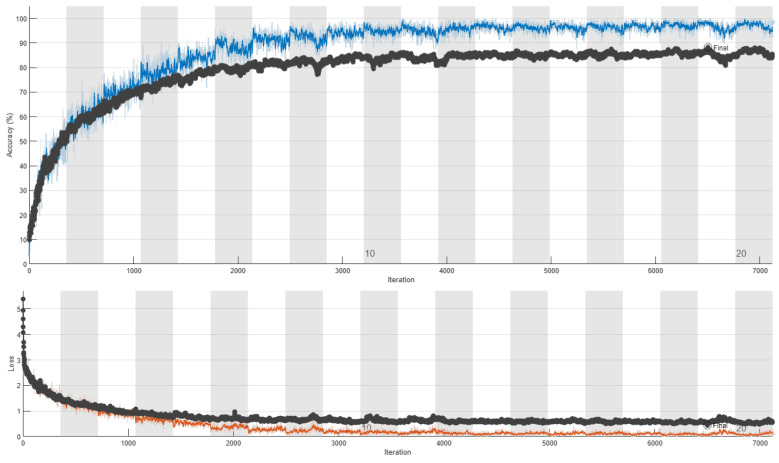
Retraining of ResNet18 without respect to humans. In the upper figure, the training accuracy is depicted in blue, while the validation accuracy is shown in black. In the bottom figure, training loss is shown in red and validation loss is illustrated in black.

**Figure 6:**
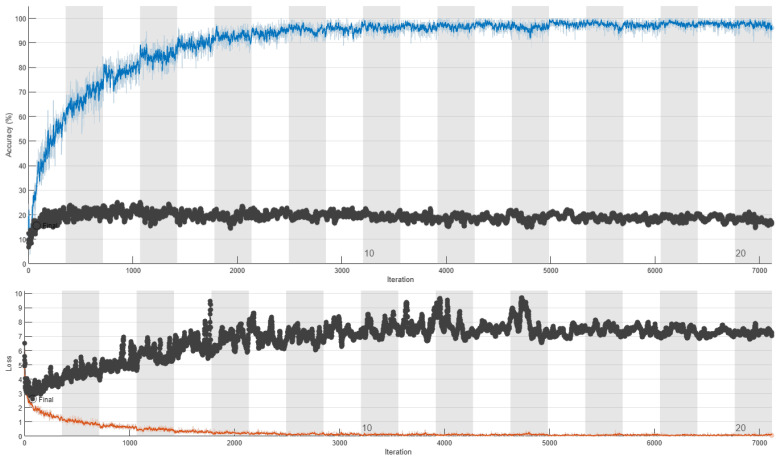
Retraining of ResNet18 with respect to humans. In the upper figure, the training accuracy is depicted in blue, while the validation accuracy is shown in black. In the bottom figure, training loss is shown in red and validation loss is illustrated in black.

The training curves of ResNet18’s retraining without respect to humans and with respect to humans are depicted in Figure 5 and Figure 6, respectively. These figures allow interesting conclusions. When the data split is conducted without respect to humans, we observe a strong correlation between training and validation accuracy, with validation accuracy closely tracking the trends of training accuracy albeit with a small difference. This consistency suggests that the model is effectively learning from the training data and generalizing well to unseen validation data. Conversely, when the data split is performed with respect to humans, a notable disparity emerges between training and validation accuracy. Despite a consistent increase in training accuracy, validation accuracy exhibits saturation, indicating that the model’s performance fails to generalize to unseen data effectively. This discrepancy suggests that the model may be overfitting to the training data when subjected to subject-based data partitioning, emphasizing the importance of proper data management practices to ensure model robustness and generalizability. The stark contrast in the behavior of training and validation accuracy highlights the critical role of data partitioning methodology in evaluating model performance accurately. These findings underscore the necessity of subject-based data partitioning to obtain reliable estimates of model generalization and mitigate the risks of overfitting.

## 5. Discussion

The empirical corroboration of data leakage in the *ImgFi* [4] WiFi CSI-based human action recognition study underscores the importance of rigorous data management practices in machine learning research [66,67]. The presence of data leakage compromises the validity and generalizability of the study’s findings. By allowing CSI images from the same individual to influence both the training and evaluation processes, the reported accuracy metrics are likely inflated, leading to an overestimation of the model’s performance. Consequently, the proposed approach may not accurately generalize to unseen data or real-world scenarios, undermining its practical utility.

Our recommendations for avoiding data leakage in WiFi CSI-based HAR are the following:

1. Subject-based data partitioning: Future studies should prioritize subject-based data partitioning to ensure the exclusivity of individuals across training, validation, and test sets. By maintaining strict isolation of subjects, researchers can mitigate the risk of data leakage and obtain more reliable performance estimates.
2. Transparent reporting: Researchers should provide detailed documentation of data partitioning procedures to facilitate reproducibility and scrutiny of the study’s methodology. Transparent reporting enables reviewers and readers to identify potential methodological flaws, such as data leakage, and assess the reliability of the reported results.
3. Publishing training curves enables other researchers to replicate and validate the presented results more effectively. By providing detailed insights into the model’s training process, a researcher can facilitate transparency and reproducibility in the field, contributing to the advancement of knowledge and best practices.
4. Reviewers play a crucial role in ensuring the integrity and reliability of published research, including identifying and addressing potential data leakage issues. Reviewers should carefully scrutinize the methodology section to ascertain how the data were partitioned for training, validation, and testing. Specifically, reviewers should look for explicit descriptions of how subjects or samples were allocated to each partition and assess whether the partitioning strategy adequately prevents information leakage between sets.
5. Publishers of publicly available databases for machine learning should consider to provide clear and comprehensive guidance on appropriate data partitioning methodologies to assist researchers in conducting robust experiments and accurately evaluating model performance. By offering recommendations for the correct train/validation/test split procedures, publishers can empower researchers to adopt best practices in data management and mitigate the risk of common pitfalls, such as data leakage. This guidance should include detailed instructions on subject-based partitioning, cross-validation techniques, and transparent reporting of data preprocessing steps to foster transparency and reproducibility in machine learning research.

Addressing data leakage is crucial for ensuring the integrity and reliability of machine learning studies. By implementing rigorous data management practices, such as subject-based partitioning and transparent reporting, researchers can enhance the validity and generalizability of their findings.

## 6. Conclusions

In this study, we conducted a critical analysis of WiFi Channel State Information (CSI) based human action recognition using Convolutional Neural Networks (CNNs), with a specific focus on addressing the issue of data leakage. Through empirical investigation and meticulous scrutiny of the methodology, experimental setup, and results presented in a notable IEEE Sensors Journal paper, we identified instances of data leakage that undermine the integrity and reliability of the reported findings. Our analysis revealed that the authors failed to implement subject-based data partitioning, leading to the inadvertent inclusion of CSI images from the same individual across the training, validation, and test sets. This critical oversight introduced a significant risk of data leakage, whereby information from the test set leaked into the training process, resulting in inflated accuracy metrics and misleading conclusions.

In conclusion, addressing data leakage is essential for advancing the field of machine learning and ensuring the reliability and generalizability of research findings. By identifying and rectifying methodological pitfalls, we can strengthen the foundations of machine learning research and pave the way for more robust and impactful applications in diverse domains. The overly optimistic performance metrics reported in studies affected by data leakage may inadvertently create a false sense of accomplishment, discouraging other researchers from critically examining the underlying methodologies and contributing to a stagnation in the advancement of the field. 

## Figures and Tables

**Table 1:** On ImageNet [60] pretrained CNNs and some of their properties. The depth is defined as the largest number of sequential convolutional or fully connected layers on a path from the network input to the network output. All models accept RGB images as input.

CNN	Depth	Size	Parameters (Millions)
ShuffleNet [57]	50	5.4 MB	1.4
VGG19 [58]	19	535 MB	144
ResNet18 [59]	18	44 MB	11.7
ResNet50 [59]	50	96 MB	25.6

**Table 2:** Dataset’s details.

Dataset Name	Action Labels	Dataset Size
WiAR [62]	two hands wave, high throw, horizontal arm wave, draw tick, toss paper, walk, side kick, bend, forward kick, drink water, sit down, draw X, phone call, hand clap, high arm wave, squat	62,415 images
Widar3.0 [64]	push, sweep, clap, slide, draw-Z, draw-N	80,000 images

**Table 3:** Parameter setting.

Parameter	Value
Dataset partitioning	Training/validation/test (0.6/0.2/0.2). Split is carried out w.r.t. humans.
Loss function	Cross-entropy
Optimizer	Adam [65] $\left(\beta_{1}=0.9,\beta_{2}=0.99,\epsilon_{2}=1\times 10^{-9}\right)$
Learning rate	0.001
Decay rate	0.8
Batch size	128
Epochs	20

**Table 4:** Comparison of percentages on WiAR.

	Reported in [4]			Retrained w/o.r.t. Humans			Retrained w.r.t. Humans		
**Architecture**	**Prec.**	**Rec.**	**F1**	**Prec.**	**Rec.**	**F1**	**Prec.**	**Rec.**	**F1**
ShuffleNet	99.4	99.4	99.4	94.5	94.5	94.3	20.4	20.0	19.9
VGG19	99.8	99.7	99.7	94.6	94.4	94.4	20.5	19.9	19.9
ResNet18	99.8	99.8	99.7	88.1	88.0	88.0	15.3	14.7	14.6
ResNet50	99.8	99.8	99.8	94.5	94.5	94.0	20.7	19.8	19.8
ImgFi	99.9	99.8	99.8	99.0	99.0	98.9	23.4	22.8	22.0

**Table 5:** Comparison of percentages on Widar3.0.

	Reported in [4]			Retrained w/o.r.t. Humans			Retrained w.r.t. Humans		
**Architecture**	**Prec.**	**Rec.**	**F1**	**Prec.**	**Rec.**	**F1**	**Prec.**	**Rec.**	**F1**
ShuffleNet	99.3	99.3	99.3	99.1	99.1	99.1	40.7	39.6	39.5
VGG19	99.8	99.7	99.6	99.7	99.7	99.6	41.0	39.5	39.8
ResNet18	99.8	99.8	99.7	97.9	97.9	97.9	30.3	29.3	29.2
ResNet50	99.8	99.8	99.8	99.3	99.2	99.2	41.4	39.6	39.4
ImgFi	99.8	99.8	99.8	99.5	99.5	99.5	47.4	45.6	43.9

## Data Availability

The data used in this study is available for download at https://github.com/linteresa/WiAR (WiAR database) (accessed on 15 March 2024 and http://tns.thss.tsinghua.edu.cn/widar3.0/ (Widar3.0) (accessed on 15 March 2024).

## Abbreviations

The following abbreviations are used in this manuscript:

CNN	convolutional neural network
CSI	channel state information
GADF	Gramian angular difference field
GASF	Gramian angular summation field
GPU	graphics processing unit
HAR	human action recognition
IEEE	Institute of Electrical and Electronics Engineers
MTF	Markov transition field
ReLU	rectified linear unit
ResNet	residual network
RP	recurrence plot
SDR	software-defined radio
SNR	signal-to-noise ratio
SVM	support vector machine
VGG	visual geometry group
